# Transperineal (TP) Versus Transrectal (TR) Prostate Biopsy: Efficacy, Safety, and Systemic Challenges—A Narrative Review

**DOI:** 10.3390/jcm15135218

**Published:** 2026-07-03

**Authors:** Piotr Szastok, Marek Doliński, Jakub Gonscz, Michał Dera, Łukasz Doliński, Mateusz Marcinek, Michał Tkocz

**Affiliations:** Department of Urology, Faculty of Medical Sciences in Katowice, Medical University of Silesia, 40-751 Katowice, Poland; s88008@365.sum.edu.pl (M.D.); s87916@365.sum.edu.pl (J.G.); s88560@365.sum.edu.pl (M.D.); s80843@365.sum.edu.pl (Ł.D.); mmarcinek@365.sum.edu.pl (M.M.); mtkocz@365.sum.edu.pl (M.T.)

**Keywords:** prostate cancer, prostate biopsy, transperineal, transrectal, cancer detection rate, complications

## Abstract

**Background**: Prostate cancer represents one of the greatest challenges in modern urology. The definitive diagnosis relies on prostate biopsy. The traditional transrectal (TR) approach carries a significant risk of infectious complications and drives antimicrobial resistance. For this reason, the transperineal (TP) approach is increasingly being considered the method of choice. This literature review aims to comparatively analyze both methods (TP vs. TR) in terms of oncological efficacy, infectious and non-infectious safety, patient-reported pain tolerance, and cost-effectiveness. **Materials and Methods**: A comprehensive search of the PubMed and Embase databases was conducted, including English-language clinical studies published after 2011. Studies directly comparing TP and TR biopsies regarding diagnostic parameters and/or complication profiles were eligible for inclusion and subsequently underwent qualitative methodological assessment. Additionally, to outline a broader background and fuller clinical context, other supplementary publications were included in the review. **Results**: Ultimately, 14 publications representing 10 unique clinical trials were included in the main analysis. The overall cancer detection rate is comparable for both methods; however, the TP approach demonstrates an advantage in detecting anterior zone lesions and—especially in combination with MRI—clinically significant prostate cancer (csPCa). Both techniques differ in their safety and tolerance profiles: TP minimizes the risk of infection and rectal bleeding but is associated with higher short-term discomfort during local anesthesia administration and more frequent hematuria. Conversely, TR is associated with prolonged post-procedural discomfort. Economic analyses yield inconclusive results depending on the time horizon adopted. **Conclusions**: The choice of biopsy technique requires a conscious clinical compromise. The TP method is becoming the preferred option due to its more favorable infectious safety profile. The development of modern urology should aim towards fully personalized diagnostics, where the decision regarding the biopsy approach is based on the patient’s best interest and is individually tailored to their anatomy, risk profile, and personal preferences.

## 1. Introduction

Prostate cancer is currently recognized as one of the greatest challenges in modern urology and oncology. It is the second most frequently diagnosed cancer globally and the sixth leading cause of cancer-related deaths among men. It is estimated that by 2040, there will be over 2 million new cases reported annually [1]. The diagnosis of this cancer, apart from a detailed medical history, physical examination, determination of prostate-specific antigen (PSA) levels, and imaging studies, rely primarily on prostate biopsy. According to the guidelines of the European Association of Urology (EAU), the definitive diagnosis of the disease always requires histopathological verification [2].

Because of this, ultrasound-guided biopsies remain the standard approach. For years, the transrectal (TRUS) route was the primary way to obtain tissue from men at high risk for prostate cancer [3]. The problem, however, is that passing the needle through the rectal wall carries a real risk of transferring intestinal bacteria directly into the prostate and the patient’s bloodstream [4]. These infections are a serious threat. They force doctors to rely on intensive antibiotic therapy, which only fuels the global crisis of antibiotic resistance and makes finding safer diagnostic options a priority [5,6,7]. Alongside severe infections like urosepsis, transrectal biopsies can also lead to major non-infectious complications such as rectal bleeding and hematuria [8]. Recognizing these dangers—especially the growing issue of drug-resistant bacteria—recent EAU guidelines recommend the transperineal (TP) approach over the transrectal (TR) route [2], and the medical community is actively debating whether this safer alternative should become our new standard of care [9]. In contrast to the classic approach, in this technique, the biopsy needle is inserted directly through the perineal skin, and the procedure itself is performed under strict ultrasound (US) guidance [10]. By bypassing the contaminated intestinal flora, this method can reduce the risk of severe infectious complications [11].

Although many publications and meta-analyses addressing this topic have appeared in medical literature in recent years, their conclusions are not always unequivocal and fully comprehensive from the perspective of daily medical practice [12,13,14,15,16]. Traditional systematic reviews, due to their rigid inclusion criteria, often narrow the analysis to isolated endpoints (e.g., exclusively the detection rate or sepsis rate). Such an approach, while statistically valuable, frequently overlooks the broader clinical context—ignoring the impact of factors such as diverse anesthesia protocols, operator learning curves, targeting technologies, or systemic and cost limitations.

In view of the above, the aim of this study is to conduct a broad, narrative comparative analysis of prostate biopsy performed via the TP and TR routes. This format allows for a multifaceted approach to the problem, going beyond a simple comparison of oncological efficacy. The paper focuses on an attempt to determine the actual risk-benefit balance of both methods. This review evaluates cancer detection rates—stratified by anatomical zones, lesion complexity, and patient subgroups—alongside a comprehensive safety profile detailing the risk and incidence of both infectious and non-infectious complications. Furthermore, it aims to compare procedural tolerance based on patient-reported outcome measures (PROMs) and pain scores, while also assessing the overall logistical and economic implications.

The inclusion of selected prospective studies alongside randomized controlled trials (RCTs) in the analysis aims to provide useful and comprehensive information, facilitating the personalization of the diagnostic process in everyday urological practice.

## 2. Materials and Methods

Search Strategy: To identify all relevant studies, we conducted a comprehensive search of the PubMed and Embase databases. We used the following combination of English keywords: (prostate biopsy OR prostatic biopsy) AND (transperineal OR TP OR “transperineal approach”) AND (transrectal OR TRUS OR “transrectal approach”) AND (complications OR infection OR sepsis OR safety OR “cancer detection” OR diagnosis OR yield OR efficacy). Our initial search, without any filters applied, yielded a total of 2831 records (957 in PubMed and 1874 in Embase). In the first screening step, we limited the articles to those published from 2011 onwards, which reduced the number to 2417 (728 from PubMed, 1689 from Embase). We selected this timeframe to focus on contemporary diagnostic standards. Furthermore, recent years have seen a documented, significant increase in the resistance of intestinal bacteria to fluoroquinolones, alongside the development of advanced imaging techniques, such as MRI-USG fusion, which have fundamentally changed how prostate biopsies are planned and performed. Subsequently, to focus on original research, we applied the “clinical trials” filter, resulting in an initial selection of 149 papers (47 from PubMed, 102 from Embase). In addition to the standard electronic database search, we manually searched the reference lists of the included publications and relevant review articles to ensure no key comparative studies were missed.

Inclusion Criteria: We included clinical trials (both randomized controlled trials and prospective studies) published in English from 2011 onwards, involving men undergoing a prostate biopsy for suspected prostate cancer. To be included, studies had to directly compare TP and TR biopsies and provide relevant data on diagnostic efficacy (such as the overall cancer detection rate, including clinically significant prostate cancer) and/or the safety profile (e.g., the incidence of infectious and non-infectious complications, pain tolerance), enabling a comprehensive comparative analysis. During the selection process, we also considered the availability of information regarding procedural time and costs, although this was not mandatory.

Exclusion Criteria: We excluded studies that evaluated only one of the methods without a comparison group; utilized other access techniques (e.g., transurethral biopsy); lacked clinical data on detection rates or complications (e.g., strictly economic models, study protocols, and registry entries without published results); did not provide access to data enabling outcome assessment; were animal studies; or were published in languages other than English.

Quality Assessment of Included Studies: Given the narrative nature of this review, we did not use the specialized risk-of-bias tools typically required for rigorous meta-analyses. Instead, we conducted a critical qualitative assessment of the included publications. We analyzed the papers primarily for study design validity (e.g., randomization, prospective nature), the adequacy and size of the study groups, the length of the follow-up period, and the clinical relevance of the endpoints. We excluded studies containing major limitations that could significantly impact the reliability of the TP vs. TR comparison from the core analysis.

Included Studies: Following the initial screening, we performed a thorough review of titles, abstracts, and full texts against our inclusion and exclusion criteria and the scope of the study. Ultimately, we included 14 scientific publications in this review, reporting the results of 10 clinical trials. We rejected the remaining papers because they failed to meet methodological requirements (such as lacking a direct comparison between both access routes), lacked separate clinical data for the TP and TR groups, had differing research objectives, were exclusively modeling-based, or met other exclusion criteria. It should be noted that the number 14 refers strictly to the primary dataset subjected to comparative analysis, whereas the complete reference list also includes supplementary literature used to outline the clinical background and facilitate a broader discussion. Furthermore, while our primary inclusion criteria focused on direct TP versus TR comparisons, we purposefully incorporated selected pivotal studies evaluating MRI-targeted techniques (such as those by Wegelin et al. and Borkowetz et al. [17,18]) into our thematic subanalyses. Although these specific studies primarily assessed targeting modalities rather than access routes alone, their inclusion provides essential clinical context regarding how advanced imaging impacts the diagnostic yield across different biopsy approaches.

During the preparation of this manuscript, the authors used Google Gemini Pro 3 (Google LLC, Mountain View, CA, USA) to improve the English language and readability. After using this tool, the authors reviewed and edited the content as needed and take full responsibility for the content of the publication.

## 3. Results

### 3.1. Key Results

To present the key scientific reports cited in this paper, Table 1 summarizes the 14 primary publications included in our review. The table details bibliographic information, cohort sizes, study designs, and key clinical conclusions. This overview allows us to evaluate the efficacy of the respective methods and track advancements in clinical standards.

#### 3.1.1. Overall Efficacy of Prostate Cancer Detection

Analysis of the research results indicates that TP biopsy frequently provides an overall diagnostic yield comparable to TR biopsy, with several studies showing no statistically significant differences in both the overall cancer detection rate (CDR) and the detection of clinically significant prostate cancer (csPCa). However, this equivalence is not absolute. Notably, evidence from the largest RCT (Bryant et al. [21]) demonstrates a higher detection rate of clinically significant prostate cancer for the TP approach. While certain data (such as Guo et al. [28]) show numerically higher detection rates for high-grade tumors (Gleason ≥ 8) using the TR route—albeit without statistical significance—the efficacy of the two access routes becomes more nuanced when a more detailed analysis of specific biopsy techniques is applied (Table 2).

#### 3.1.2. Influence of Malignant Tumor Localization Within the Prostate Gland on Detection Efficacy

Based on the table, it can be concluded that prostate cancer detection efficacy varies depending on the lesion’s localization and the biopsy access route. TP biopsy shows significantly higher efficacy in detecting cancer in the anterior zone of the prostate gland compared to TR biopsy. However, for lesions located in the apex and base of the prostate gland, no statistically significant differences in detection efficacy were observed between the two methods. Furthermore, in the peripheral and transitional zones, although TP biopsy provides better quality of retrieved material (longer cores) and a higher detection rate (CDR), these differences did not reach statistical significance compared to TR biopsy (Table 3).

#### 3.1.3. Influence of the Applied Biopsy Type and Access Route on Prostate Cancer Detection Efficacy

It should be noted that the efficacy of prostate cancer (PCa) detection, especially its clinically significant forms (csPCa), varies depending on the type of biopsy used. TP biopsy, particularly when combined with image guidance (fusion or combined systematic and targeted), may offer enhanced diagnostic value over TR biopsy in detecting csPCa, as confirmed by statistically significant differences in several studies. For targeted biopsy alone (e.g., fusion), the access route (TP vs. TR) may not have a significant impact on detection efficacy. Studies also show that a comprehensive approach, combining systematic and targeted biopsy, is significantly more effective than using only one of these biopsy types. Furthermore, although systematic biopsy performed via the TP route may lead to a higher number of positive cores, this does not always translate into a statistically significant advantage in the overall cancer detection rate compared to TR systematic biopsy (Table 4).

#### 3.1.4. Detection Efficacy Depending on Belonging to a Specific Patient Subgroup

Although the results are somewhat varied, it can be observed that in “biopsy-naive” patients (those without prior biopsies), especially in the context of detecting clinically significant cancer, TP biopsy appears to show superiority or at least comparable efficacy to TR biopsy, and in some studies, this advantage was statistically significant. It is also clear that the use of MRI to guide biopsies (targeted biopsies) improves detection efficacy, particularly for early, difficult-to-localize lesions (Table 5).

### 3.2. Safety Profile

#### 3.2.1. Infectious Safety

Data in Table 6 indicate a high infectious safety profile for both methods; while some studies show a clear numerical advantage in favor of TP, it remains below the threshold of statistical significance [21,23,25]. The exception is the TRANSLATE study, which demonstrated a significant advantage of the TP access in preventing infections in a long-term follow-up (4 months) [21].

#### 3.2.2. Non-Infectious Complications

The overall frequency of non-infectious complications is similar for both techniques; however, they present different adverse event profiles. TR access is associated with a significantly higher risk of blood in the stool, as confirmed by TRANSLATE, Waisman Malaret et al. and Guo et al. [21,28,30], whereas patients undergoing TP biopsy more frequently experience hematuria (particularly after 24 h) and voiding disorders [29,30]. The incidence of acute urinary retention (AUR) and blood in semen remains comparable between both groups across analyzed studies [19,20,21,23,24,25,29,30,31] (Table 7).

#### 3.2.3. Pain Tolerance and Quality of Life

Available data regarding patients’ subjective experience indicate a varied pain profile depending on the chosen biopsy technique, with key differences relating to specific stages of the procedure. In the controlled study by Cerruto et al., no significant differences were found in the overall intensity of pain during the procedure; mean Visual Analog Scale (VAS) values were similar (1.56 ± 1.73 for TR vs. 1.42 ± 1.37 for TP; *p* = 0.591) [19]. Schieda et al. reached similar conclusions, showing no significant differences in post-procedure pain scores (median 2 [1,2,3,4,5] for TR vs. 3 [2,3,4] for TP; *p* = 0.09) [20]. The PERFECT project yielded consistent results, where average pain level increased by 1 point immediately after biopsy in both groups and gradually subsided over following days, showing no differences between TR and TP groups [24]. Similarly, Tricard et al., in a study involving two biopsy series, reported no differences in quality of life or pain intensity between TR and TP groups [23].

However, significant differences in pain perception were noted in large multicentered studies. In the TRANSLATE study, TP biopsy was perceived as more problematic immediately after the procedure (38% vs. 27%; *p* < 0.001). Patients after TP biopsy more frequently reported pain, discomfort and embarrassment associated with the procedure as occurring “often”. Interestingly, on day 7 post-procedure, it was the TR group that reported more complaints (25% vs. 18%; *p* = 0.0063). No differences were found in overall quality of life measured by EQ-5D and VAS tools [21]. In the PREVENT study, mean pain during the procedure was higher in the TP group (3.6 vs. 3.0) and the percentage of patients reporting severe pain (≥7) was nearly twice as high in this group (12% vs. 7%; *p* = 0.035). Nevertheless, after 7 days, TR patients more often reported persistent symptoms (14% vs. 9.7%; *p* = 0.08) [25].

A similar relationship was observed by Guo et al. [28], where among 328 analyzed patients, the average VAS pain score during the procedure was significantly higher in the TP group. Detailed analysis showed that in the TP approach, pain was most severe during local anesthesia administration (63.6% vs. 17.5%; *p* < 0.001), while in the TR group, the most troublesome part was the insertion of the ultrasound probe (42.2% vs. 14.5%; *p* < 0.001). Post-procedure complications in the form of mild pain occurred significantly more often in the TP group (35.3% vs. 13.0%; *p* < 0.001) [28]. Procedural differences were also confirmed by Berquin et al. [29]. Although probe placement generated pain of 1 VAS score in both groups, local anesthesia of the prostate was significantly more painful in the TP access (4 vs. 2; *p* < 0.01). Conversely, the collection of specimens tended to be less painful in the TP group (1 vs. 2) (*p* = 0.06) [29]. Waisman Malaret et al. [30], using a questionnaire answered by nearly 96% of participants, showed that pain after the procedure (on the evening of the biopsy day and on day 3) was significantly higher and more frequently reported by TP patients (61.0% vs. 49.3%; *p* = 0.0012 and 21.2% vs. 14.6%; *p* = 0.022, respectively). In this cohort, anesthesia also generated higher pain (3.3 vs. 0.7) (*p* < 0.001), while probe insertion was worse tolerated in the TR group (2.3 vs. 1.8) (*p* < 0.001) [30].

### 3.3. Economic Analysis of Both Methods

The TP biopsy and the TR biopsy differ in terms of the cost of the procedure itself and the potential cost of treating post-procedural complications. These differences raise questions regarding the cost-effectiveness of each method in relation to the patient’s health benefits [33]. These elements of the cost-effectiveness problem are reflected in the ICER (Incremental Cost-Effectiveness Ratio), which measures the relationship between the difference in costs of the two methods and the health effect expressed in QALY (Quality-Adjusted Life Year) [33].

The present analysis included the PREVENT-based economic simulation by Huang et al. and the TRANSLATE within-trial economic analysis by Little et al. [22,26]. The Huang et al. study was a model-based cost-effectiveness analysis derived from PREVENT data, whereas the TRANSLATE economic analysis by Little et al. was conducted alongside the TRANSLATE randomized controlled trial reported by Bryant et al. [22,26]. The TRANSLATE economic analysis extends and complements earlier findings from the Wilson et al. economic evaluation, providing a broader and more up-to-date assessment of the cost-effectiveness of TP and TR methods [26,34].

#### 3.3.1. Huang et al. [26] PREVENT-Based Economic Simulation

The analysis by Huang et al. was a PREVENT-based economic simulation conducted in the United States [26]. It should be distinguished from the clinical PREVENT randomized trial, as the present section refers to the cost-effectiveness model rather than to the clinical trial itself.

The economic analysis was performed over a 7-day time horizon for a cohort of 1000 healthy patients aged 50–70 years, using a Markov model. The model divided patients into the following states: “Healthy”, “Infected”, “Urinary retention”, “Combined”—meaning both infection and urinary retention—and “Recovered”.

Patients in the “Healthy” state passed through simulated cycles of the listed states for both TP and TR methods in order to calculate the total costs associated with each method. A summary of the costs and parameters typical for the defined states in TP and TR is presented in Table 8. All costs related to the procedure and its potential complications were considered from the payer’s perspective, appropriate for the United States healthcare system in 2024.

Utility parameters were also assigned to each state, and the QALY was calculated. Subsequently, an incremental analysis was performed, taking into account the difference in costs and the difference in health effects expressed in QALY, which formed the basis for calculating the ICER and identifying the dominant strategy.

The main factors differentiating the costs of TP and TR in the Huang et al. [26] model were the costs of rectal swab testing and antibiotic prophylaxis in the TR strategy, as well as the assumed probability of infection, estimated at 1.6% for TR and 0% for TP over the 7-day analysis horizon. Therefore, the modeled economic advantage of TP in this simulation should be interpreted with caution, as it was strongly influenced by the predefined mathematical assumption of no infectious complications in the TP group. In turn, the TP method generated higher costs of disposable materials. In the final calculation, the total cost of TP was 83.62 USD lower than that of TR, while the health effect expressed in QALY was slightly higher for TP. Thus, in the base-case model, TP was classified as the dominant cost-effective strategy [26].

Limitations of the analysis:

- a short, 7-day time horizon covering only direct complications after prostate biopsy;
- the assumed infection probability of 0% for the TP method;
- the absence of sepsis in the base-case analysis, despite its inclusion in the model;
- the reliance of the model on assumptions regarding complication rates, which may differ across other populations and healthcare systems.

#### 3.3.2. TRANSLATE Within-Trial Economic Analysis by Little et al. [22]

Unlike the Huang et al. PREVENT-based economic simulation, the TRANSLATE within-trial economic analysis by Little et al. [22] was not a purely model-based analysis. It was conducted alongside the TRANSLATE randomized controlled trial reported by Bryant et al. in the United Kingdom, from the perspective of cost-effectiveness for the National Health Service (NHS) [22,26]. A total of 1126 randomly assigned biopsy-naïve men were included in the trial. The TP biopsy group (n = 562) and the TR biopsy group (n = 564) were monitored for 4 months after the procedure. Health outcomes were described using accumulated quality-adjusted life years (QALYs). In addition to healthcare system costs, the analysis included patient-specific costs, such as out-of-pocket expenses, “time off work”, and “time off usual activities”. The economic and health-related data included in the analysis (Table 9) were reported by Little et al. and are based on 2022 costs [22].

In the TRANSLATE economic analysis, over a 4-month observation horizon, the TP method was associated with a higher total healthcare cost compared with TR (+£149; 95% CI: £61–£236; *p* = 0.001), while also showing a slightly lower health effect expressed in QALY (−0.004; 95% CI: −0.009 to 0.001; *p* = 0.098). In the incremental analysis, TP was dominated by TR, indicating no cost-effectiveness advantage under the conditions of the NHS system [22].

Limitations of the analysis:

- a relatively short, 4-month observation horizon, which may not capture long-term clinical and economic consequences;
- the analysis was conducted from the NHS perspective, which limits the direct transferability of the results to other healthcare systems;
- the lack of a full, detailed breakdown of all components contributing to the total cost reported in the study.

## 4. Discussion

A prostate biopsy is essential for a definitive diagnosis of prostate cancer [4]. This step is crucial because the pathology findings dictate the immediate treatment plan and ultimately influence the patient’s long-term health. This is especially true for men with low-risk tumors, where active surveillance and focal therapy offer real alternatives to avoid aggressive radical treatment [23].

Despite this, current medical literature still lacks a definitive answer on which of the two main approaches—the traditional TR or the newer TP route—is objectively better and should become the standard of care [12,13,14,15,16]. When we look at the multicenter randomized clinical trials analyzed in this review, such as PREVENT, TRANSLATE, PERFECT, or ProBE-PC [21,24,25,26,30,31,32], neither method shows clear-cut superiority. However, the data does reveal certain trends that point to a necessary clinical compromise. The two techniques differ significantly, not just in their oncological efficacy, but also in their safety profiles, cost-effectiveness, and how well patients tolerate them.

The ongoing debate regarding the optimal prostate biopsy route is reflected in the current international guidelines, which demonstrate different recommendations. According to the European Association of Urology (EAU) guidelines, the TP approach is preferred over the TR route. This recommendation is primarily driven by the growing importance of antibiotic stewardship and the strict European regulatory conditions that have suspended the perioperative prophylactic use of fluoroquinolones. For the EAU, the comparable cancer detection rates and the feasibility of performing TP biopsies under local anesthesia justify a procedural shift to minimize infectious risks.

The American Urological Association (AUA) [35] guidelines maintain a more conservative approach, considering both routes clinically acceptable. The AUA emphasizes that recent large-scale randomized controlled trials, such as the PREVENT and ProBE-PC studies, have not demonstrated a definitive superiority of the TP approach in terms of either reducing infectious complications or improving the detection of clinically significant prostate cancer. Consequently, the AUA notes that it is difficult to exclusively recommend the TP route at this time. Instead, they state that TR biopsies remain an appropriate option, provided that risk-reducing measures—such as culture-directed targeted therapy via rectal swabs or augmented antibiotic prophylaxis—are strictly implemented. Furthermore, the American guidelines acknowledge the practical and logistical challenges associated with transitioning to the TP approach, including the need for specific equipment, such as biplanar probes, and specialized training in perineal local anesthesia delivery. Ultimately, this transatlantic difference illustrates two distinct strategies: the EAU prioritizes a systematic bypass of the rectal flora, while the AUA balances the clinical outcomes of both techniques with healthcare logistics and available prophylactic measures.

It is worth noting that the noticeable variations in reported outcomes—both for efficacy and safety—likely stem from methodological differences across the studies. The main challenge in comparing these results directly is the lack of standardized protocols.

This is particularly obvious regarding anesthesia. For instance, in the TRANSLATE [21] and PREVENT [25,26] trials, clinicians performed both TR and TP procedures exclusively under local anesthesia in outpatient settings. In contrast, Tricard et al. [23] compared traditional TR biopsies under local anesthesia with TP biopsies performed under general anesthesia. Meanwhile, the PERFECT trial [24] used the type of anesthesia, local versus general, as a criterion to evenly divide patients into study groups. The FUTURE trial [18] routinely performed its TP biopsies, FUS-TB, under general or spinal anesthesia. These differences have real practical implications because the type of anesthesia directly affects how a patient perceives pain and whether they report other postoperative issues.

Another variable affecting the outcomes was the different approaches to targeting MRI-visible lesions. Many studies, including PREVENT [25,26], PERFECT [24], and the analysis by Schieda et al. [20], used dedicated software-assisted fusion. Conversely, the multicenter TRANSLATE trial [21] relied solely on cognitive targeting. Taking a different angle, the FUTURE trial [18] directly investigated the differences between cognitive fusion, software fusion, and in-bore MRI biopsies.

Finally, differing follow-up periods also contributed to the varying complication rates and pain scores. For example, Berquin et al. [29] focused on early assessment, evaluating patients at 12, 24, and 48 h post-biopsy. The PREVENT trial [25,26] used a slightly longer window, relying on 7-day questionnaires, whereas the TRANSLATE trial [21] tracked patients through multiple stages up to 4 months after the procedure.

Numerous clinical studies comparing TR and TP biopsy in the context of overall prostate cancer (PCa) and clinically significant prostate cancer (csPCa) detection present heterogeneous results, which complicates the formulation of unequivocal conclusions. Some studies do not report statistically significant differences in the efficacy of both access routes. For instance, the study by Mian et al. [32] showed comparable csPCa detection rates for both methods, but without achieving statistical significance. Similarly, Cerruto et al. [19] in their randomized study found no significant difference in the overall cancer detection rate (CDR) between TR and TP (*p* = 0.846). Converging observations were made by Ploussard et al. [24], who found no significant differences in the detection of either csPCa or PCa of any grade. The analysis by Le Hang Guo et al. [28] also confirms the lack of statistical significance for overall PCa detection and the percentage of positive cores, suggesting that in some patient populations and with specific protocols, both access routes may offer similar efficacy.

On the other hand, some literature leans towards the superiority of the TP approach, especially in specific contexts. A “within-person” study by Yaary Ber et al. [27], where both methods (TR-fusion and TP-fusion) were applied to the same patients, revealed a statistically significant advantage of TP-fusion in csPCa detection (*p* = 0.029). The same phenomenon was observed by Thibault Tricard et al. [23], where TP biopsy significantly outperformed TR biopsy in detecting csPCa (*p* = 0.019). Richard J. Bryant et al. also provided evidence of a statistically significant advantage of the TP method in detecting higher-grade cancer (GGG ≥ 2). These discrepancies may result from differences in study protocols, including the number of cores taken, targeting techniques, patient populations, and diagnostic criteria adopted, which underscores the need for further standardization and clarification of comparative conditions.

Analysis of studies suggests that fusion biopsies (FusPbx) or combined biopsies (comPbx, a combination of FusPbx and systematic biopsy–SysPbx), integrating mpMRI images with real-time ultrasonography, may offer better detection rates, especially for csPCa. A prospective cohort study by Angelika Borkowetz et al. [17] showed that combined biopsy (comPbx) achieved the highest detection efficacy for csPCa and PCa, significantly outperforming single methods (*p* < 0.005). A key conclusion from this work is also that patients with a high PI-RADS score (4–5) benefited most from combined biopsy, with csPCa detection reaching 61% (*p* < 0.005). This emphasizes that in patients with clearly suspicious lesions on mpMRI, a combined strategy, likely due to the complementarity of methods (targeting foci and systematically sampling the entire gland), is the most effective.

In contrast, the study by Ploussard et al. [24] on “biopsy naive” patients did not show significant differences between targeted TR and TP biopsy in detecting csPCa or overall PCa. This discrepancy may suggest that in the population of patients without previous biopsies, the influence of the access route itself may be less dominant than in cases involving repeated procedures or specific lesion locations.

The localization of suspicious lesions in the prostate gland plays a crucial role in choosing the optimal access route. This is clinically relevant because systematic TR biopsy may miss a proportion of clinically significant tumors, particularly those located in the anterior and apical regions of the gland [36]. Older studies, such as those cited by Cerruto et al. [19], already suggested the superiority of TP biopsy in detecting cancer in the transitional zone (TZ) and anterior zone, which is an area more difficult to access for TR biopsy. Newer data also confirm this: Nicola Schieda et al. [20] demonstrated a significant advantage of TP biopsy in detecting csPCa in the anterior zone (*p* = 0.03).

In the study by Guillaume Ploussard et al. [24], a statistically significant advantage of the TR method was noted in detecting cancer in posterior lesions (*p* = 0.044). This aligns directly with anatomical proximity, as the posterior zone is immediately adjacent to the TR ultrasound probe, allowing for optimal sampling trajectories.

Interesting conclusions also come from the study by Maria Angela Cerruto et al. [19], which, despite the lack of significant differences in overall CDR, showed that TP biopsy allowed for the retrieval of significantly longer cores in the apex and transitional zone. Although this objective improvement in biopsy quality (which may be important for the pathologist evaluating the material) did not translate into a globally higher cancer detection rate in this particular study, it may be relevant in the context of more precise grading or tumor volume assessment. In this context, precise pre-procedural determination of prostate volume (PV) remains vital; while traditional methods like digital rectal examination (DRE) and TRUS frequently under- or overestimate gland dimensions, multiplanar MRI-based measurements provide a significantly more accurate approximation of the actual prostate size compared to radical prostatectomy specimen weights, facilitating optimal risk stratification and treatment planning [37]. Olivier Wegelin et al. [18], analyzing various variants of targeted biopsy, did not find significant differences in csPCa detection efficacy across different zones (anterior, posterior, peripheral, transitional), which may suggest that advanced targeting techniques, regardless of the specific TR implementation, are effective in various regions of the gland.

Based on a comprehensive analysis of the presented studies, identifying one universally “best” access route guaranteeing the highest prostate cancer detection rates across all scenarios is challenging. The scientific literature provides evidence for both overall equivalence and distinct, route-specific advantages depending on clinical variables. However, the following evidence-based conclusions can be formulated. First, in biopsy-naïve patients without targeted mpMRI lesions, the overall efficacy of both access routes is comparable; the lack of statistically significant differences in broad cohorts [19,24,28,32] indicates that for standard systematic sampling, neither method is oncologically superior. Second, for lesions located in the posterior zone, the TR approach demonstrates superior diagnostic efficacy. Rather than being an unexplained phenomenon, this aligns directly with anatomical proximity, as the posterior peripheral zone lies immediately adjacent to the TR ultrasound probe, allowing for direct and highly efficient needle trajectories as confirmed by the sub-analyses in study [24].

The fundamental importance of magnetic resonance imaging (mpMRI) as a biopsy qualification examination should also be emphasized. As shown by the PROMIS study [38], mpMRI is characterized by very high sensitivity in detecting clinically significant cancer, with moderate specificity (41%). In comparison, traditional systematic TRUS biopsy in the same study showed significantly lower sensitivity (48%), which was associated with a high risk of missing serious disease. This unequivocally indicates that mpMRI imaging should be an indispensable diagnostic step, regardless of the ultimately chosen access route (TP or TR). Furthermore, comparative data evaluating pre-biopsy mpMRI and combined fusion-targeted/systematic biopsy protocols against definitive post-radical-prostatectomy specimen histology confirm high concordance rates for both lesion laterality and Gleason scores, reinforcing the clinical value of mpMRI in accurately staging and mitigating the underestimation of clinically significant PCa [39].

To overcome the baseline limitations of conventional TRUS and provide a high-resolution alternative or supplement to current mpMRI-centric pathways, micro-ultrasound (MUS) technology has recently emerged as a promising real-time targeting system [40]. By utilizing high-frequency transducers (up to 29 MHz), providing up to a 300% enhancement in spatial resolution compared with standard ultrasound equipment, MUS allows clinicians to dynamically visualize subtle micro-architectural tissue variations in real time [40]. Being native to the ultrasound platform itself, MUS functions completely independently of the selected approach method and thus serves as a versatile tool for real-time biopsy either as a standalone diagnostic system or as a complementary framework to guide targeted sampling via both TR and TP approaches, thereby moderating the logistical, economic and software complexities typically associated with software-assisted MRI fusion [40].

In the aspect of safety, the analysis of the collected evidence indicates fundamental differences in the safety profiles of both methods, especially in the context of the growing global problem of antibiotic resistance, where antimicrobial stewardship becomes crucial. While traditional TR access achieves low infection rates, it does so only at the cost of aggressive antibiotic prophylaxis. In an era of spreading multi-drug resistant (MDR) E. coli strains, such as the highly virulent ST131 clone [41], this becomes a risky strategy. Studies confirm that the resistance of rectal flora to fluoroquinolones is difficult to predict based on patient’s history alone [42], and the presence of MDR strains is a direct risk factor for hospitalization after a TR procedure [43], prompting the use of potent drugs like ertapenem [44], or procedures based on rectal swabs [45]. The advantage of TP biopsy lies in the drastic reduction in infectious complications and sepsis while allowing the avoidance of antibiotics, as confirmed by the TRANSLATE [21] study and meta-analyses by Marra et al. [12], Yang et al. [13] and Calpin et al. [15], documenting the superior infectious safety of the TP method. Nevertheless, publications such as the meta-analysis by Suartz et al. [14], or studies by Schieda et al., Berquin et al., Mian et al. [20,29,31] and the multicenter PREVENT trial [25] point to a high safety profile for both methods with no statistically significant differences in some cohorts.

In the area of non-infectious complications, there is a clear distinction in the complication profiles. The TP method shows an advantage in reducing the risk of major bleeding, as the perineum lacks hemorrhoidal plexuses and any bleeding can be easily controlled by compression. While blood in the stool occurs significantly more often after the TR biopsy [21,28,30], which is also confirmed by the meta-analysis by Du et al. [16], the TP group more frequently reports transient hematuria and voiding disorders [29,30]. Although some sources suggest a higher risk of Acute Urinary Retention (AUR) in the TP group [16], most analyses, including studies by Tricard et al., PERFECT, Mian et al. [23,24,31] and the meta-analysis by Calpin et al. [15], find no significant differences in AUR incidence. Conversely, Cerruto et al. point to an identical overall complication rate in both groups [19].

Choosing between TR and TP biopsy consequently involves a clear differentiation of the safety profile despite a similar complication rate. The TP method represents a safer alternative regarding hemorrhagic risk (lower incidence of blood in stool and easier control of potential bleeding), albeit at the cost of a transient increase in urinary tract symptoms such as hematuria or dysuria. Simultaneously, the lack of unequivocal evidence for an increased risk of AUR after TP biopsy allows this method to be considered clinically comparable to TR in terms of obstructive risk while maintaining a more favorable vascular profile.

Regarding patient-reported outcomes, the choice between access routes involves a clear procedural and temporal trade-off. While larger trials report higher intraprocedural pain for TP biopsy—primarily during perineal local anesthesia administration—TR biopsy is associated with worse tolerance during ultrasound probe insertion and more prolonged post-procedural discomfort extending to day seven. However, long-term health-related quality of life remains comparable between both techniques, indicating that the acute, short-term discomfort of the TP approach represents an acceptable clinical compromise for a significantly optimized safety profile.

In addition to safety and procedural tolerability, economic aspects remain an important criterion for selecting the biopsy method. However, the cost-effectiveness assessment of TP and TR is not straightforward and largely depends on the adopted analytical assumptions and the context of the study. The discrepancy between the Huang et al. PREVENT-based economic simulation and the TRANSLATE within-trial economic analysis by Little et al. shows that the same intervention may be assessed differently depending on the analytical model, length of follow-up, and healthcare system [22,26].

In the Huang et al. [26] PREVENT-based model, the favorable economic result for TP should be interpreted with caution because it was derived from a short-term simulation that was highly sensitive to assumptions regarding early infectious complications, particularly the predefined 0% infection risk in the TP group. This assumption substantially reduced infection-related costs assigned to TP and influenced the final cost-effectiveness result. The 7-day time horizon further limited the analysis to early post-procedural events and reduced the ability to assess longer-term clinical and economic consequences [26].

By contrast, the TRANSLATE economic analysis by Little et al., based on patient-level economic data collected alongside the TRANSLATE randomized controlled trial reported by Bryant et al., provides a more direct estimate of procedure-related costs and health effects and does not confirm the cost-effectiveness advantage of TP [22,26]. Over a longer, 4-month follow-up period, TP was associated with higher costs and no significant QALY gain, meaning that TP was dominated by TR in the incremental analysis [22]. This suggests that, in a trial-based clinical setting and when broader healthcare costs are taken into account, the economic benefits of TP may be less evident than indicated by short-term model-based analyses.

The time horizon remains a key factor influencing the differences between the analyses. Extending the follow-up period reduces the relative importance of rare acute complications, such as severe infections, while increasing the impact of procedural and organizational costs. This means that short-term models may overestimate the role of infectious complications in assessing the cost-effectiveness of the procedure.

The healthcare system context is also important, as differences between the United States system and the British NHS influence cost structures, reimbursement mechanisms, financing of healthcare services, availability of resources, organization of biopsy pathways, and management of complications. Consequently, the findings of the Huang et al. [26] and Little et al. [22] economic analyses cannot be directly generalized to all healthcare settings. Overall, the current economic assessment of TP and TR biopsy remains preliminary and relatively limited, being based mainly on selected analyses conducted within specific national systems. A full evaluation of the economic value of both biopsy approaches would require longer follow-up, more detailed and transparent cost reporting, and dedicated Health Technology Assessment (HTA) analyses adapted to specific healthcare systems.

Our review has several notable strengths. We conducted a thorough search of major databases (PubMed and Embase) to identify relevant studies. Unlike many previous papers, we did not limit our scope solely to clinical efficacy and safety profiles. We also evaluated the cost-effectiveness and logistical challenges associated with both access routes.

We must also acknowledge our study’s limitations. As discussed earlier, the clinical trials included in our main review are methodologically heterogeneous—particularly regarding patient populations (e.g., biopsy-naïve vs. prior-negative), the anesthesia used, targeting techniques, and patient follow-up times. Because of this profound variation, and the narrative design of our review, we did not perform a statistical meta-analysis. Consequently, we could not calculate pooled odds ratios (ORs) or quantify the absolute differences in csPCa detection rates and infection risk. While we attempted to provide stratified observations where the original data allowed (e.g., analyzing detection efficacy by biopsy type and patient subgroups in Table 4 and Table 5), the lack of exhaustive stratified analyses and validated risk-of-bias tools represents a standard limitation inherent to narrative reviews. To provide greater transparency regarding the quality of the analyzed evidence, we have summarized the key methodological limitations of each primary study in Table 1. Furthermore, it is critical to state that this review focuses strictly on periprocedural complications and short-term diagnostic parameters. This work does not evaluate the long-term clinical or oncological impacts of the selected method, such as its influence on active surveillance, subsequent histological reclassification rates, or overall prognosis. Ultimately, to overcome these boundaries, future investigations should be developed in accordance with established methodological standards, such as the PRISMA guidelines, the Cochrane Handbook, and AMSTAR 2. The adoption of such systematic methodologies is essential to enable a rigorous quantitative assessment of evidence quality and further improve the reproducibility of the study selection process.

Despite the valuable clinical data currently available, there is still a clear need for broader, multicenter trials. For upcoming research to be truly impactful, it must be built on strict methodological frameworks. Future trials must adopt consistent approaches to anesthesia and imaging, along with a universal system for recording patient discomfort and adverse events. Pairing this level of standardization with long-term clinical and economic follow-up is essential to establishing a definitive care pathway. At its core, modern prostate diagnostics must be patient-tailored—treating a man’s individual anatomy, risk factors, and personal choices as equally important to the procedural yield.

## 5. Conclusions

Our findings show that choosing a prostate biopsy technique is not about making a blanket recommendation for one method over the other. Meta-analyses (including Marra, Suartz, Yang) confirm that the TP and TR approaches demonstrate equivalent overall prostate cancer detection rates. While some trials (e.g., Ber, Tricard, and the TRANSLATE study) report higher diagnostic efficacy for TP, these represent exceptions driven by specific study designs—such as variations in anesthesia protocols—rather than a general trend, as the true advantages of the TP route remain its superior sampling access to the anterior and transition zones and lower infection risk. In an era of rising antibiotic resistance, this gives the TP route particular clinical significance.

Still, practical issues like cost, logistics, and pain tolerance keep the TR method relevant in daily practice. Clinicians must weigh the specific clinical context for each patient—including mpMRI findings, previous biopsies, and distinct risk factors—to select the most appropriate route.

We must view modern prostate biopsies as highly individualized procedures. The best diagnostic strategy is a deliberate compromise—balancing maximum safety and patient comfort against the available healthcare resources.

## Figures and Tables

**Table 1:** Summary of the included studies comparing TP and TR prostate biopsy.

No.	Study (Author, Country, Year)	Study Design	Cohort Size (TR/TP)	Key Conclusions	Methodological Limitations
[19]	Cerruto et al., Italy, 2014	RCT	54/54	Comparable overall CDR and safety profiles; similar pain tolerance.	Single-center design; operator bias (2 operators: 1 dedicated strictly to TP and 1 to TR).
[20]	Schieda et al., Canada, 2025	RCT	114/119	Comparable overall CDR; TP advantageous for diagnosing challenging lesions.	Short follow-up for complications (1–2 weeks); variable number of targeted cores per lesion (operator-dependent).
[21]	Bryant et al., UK, 2025	RCT	564/562	Largest RCT; superior diagnostic efficacy and infectious safety for TP.	Lack of blinding (open-label design); reliance exclusively on cognitive targeting rather than software-based MRI-US fusion.
[22]	Little et al., UK, 2026	RCT	564/562	Higher procedural costs for LATP with no significant short-term QALY benefit over TRUS.	Short 4-month follow-up; UK/NHS-based cost data limiting generalisability.
[23]	Tricard et al., France, 2025	RCT	45/45	Safe and effective; increased CDR in repeat MRI-guided biopsies.	Single-center, open-label design; major procedural heterogeneity (e.g., local vs. general anesthesia); different antibiotic protocols.
[24]	Ploussard et al. (CCAFU-PR1), France, 2024	RCT	128/122	Comparable overall clinically significant CDR and safety; TR superior for posterior lesions; longer procedural time for TP.	Open-label (unblinded) design; lack of standardization for systematic biopsies (performed at operator’s discretion); short follow-up for functional outcomes (15 days).
[25]	Hu et al., USA, 2024	RCT	280/287	Comparable clinically significant CDR and infectious safety	Open-label (unblinded) design; short follow-up period for complications (7 days); inconsistent informed consent procedures across study sites.
[26]	Huang et al., USA, 2024	RCT	280/287	Office-based TP without prophylaxis avoids rectal cultures and is highly cost-effective.	Secondary model; 2-week time horizon; lacks long-term clinical outcomes; limited health-system generalizability.
[27]	Ber et al., Israel, 2020	RCT	77	TP biopsy superior for clinically significant CDR in MRI-visible lesions.	Within-subject & single-operator design; systematic biopsies performed exclusively via TP.
[28]	Guo et al. China, 2015	RCT	166/173	Comparable overall CDR; lower complication rates, but TP less well-tolerated.	Open-label design; major sampling bias (TZ explicitly omitted in TR arm); 7-day follow-up.
[29]	Berquin et al., Belgium, 2023	Prospective cohort	61/67	CDR not evaluated. Comparable overall complications and tolerability. Trade-offs: more pain during anesthesia and hematuria for TP; early rectal bleeding for TR.	Observational design with selection bias; unequal core counts; 48 h follow-up
[30]	Waisman M. et al., USA, 2026	RCT	384/398	TP is associated with greater pain during anesthesia, transient urinary dysfunction, and decreased QoL	Subjective nature of PROMs (NRS, IPSS); short functional follow-up (2 weeks); lack of objective functional parameters (e.g., uroflowmetry) to corroborate symptom scores.
[31]	Mian et al., USA, 2024	RCT	351/367	Comparable infectious and non-infectious complications; no cases of sepsis in either group; both approaches safe and viable.	Single-center design limits generalizability; severe racial homogeneity (93% White participants).
[32]	Mian et al., USA, 2024	RCT	384/398	Comparable overall and clinically significant CDR.	Single-center design; severe racial homogeneity (92.6% White participants) limits ethnic generalizability; coin-flip randomization.

Abbreviations: CDR, cancer detection rate; IPSS, International Prostate Symptom Score; LATP, local anaesthetic transperineal; MRI, magnetic resonance imaging; NHS, National Health Service; NRS, numerical rating scale; PROMs, patient-reported outcome measures; QALY, quality-adjusted life year; QoL, quality of life; RCT, randomized controlled trial; TP, transperineal; TR, transrectal; TRUS, transrectal ultrasound; TZ, transitional zone; US, ultrasound.

**Table 2:** Comparison of prostate cancer detection efficacy for TR and TP approaches.

Study (Ref No.)	Patient Subgroup	Transrectal Efficacy	Transperineal Efficacy	Comment
Badar M. Mian et al. [32]	Overall (all patients)	47% clinically significant PCa	43% clinically significant PCa	No information of statistical significance
	MRI-positive	56% clinically significant PCa	53% clinically significant PCa	N/A
	MRI-positive, biopsy-naïve	59% clinically significant PCa	62% clinically significant PCa	N/A
	Anterior tumors on MRI	44.5% clinically significant PCa	39% clinically significant PCa	N/A
	MRI-negative	16% clinically significant PCa	14% clinically significant PCa	N/A
Angelika Borkowetz et al. [17]	Biopsy-naïve patients	Systematic biopsy clinically significant PCa: 35%	Fusion biopsy clinically significant PCa: 38%	Combined biopsy (Systematic + Fusion) efficacy = 44%; Fusion vs. Systematic: *p* = 0.296 (Not Significant); Combined vs. single: *p* < 0.005 (significant)
Guillaume Ploussard et al. [24]	Biopsy-naïve patients	Significant PCa: 54.2%, Overall detection rate: 64.1%	Significant PCa: 47.2%, Overall detection rate: 71.3%	*p* = 0.6235 (clinically significant cancer, ISUP ≥ 2 detection); *p* = 0.2209 (any grade detection)
Le Hang Guo et al. [28]	Biopsy-naïve patients	Detection rate (overall): 35.3%	Detection rate (overall): 31.3%	No significant statistical difference between methods; *p* = 0.566
		Gleason ≤ 6: 10.4%	Gleason ≤ 6: 10.8%	*p* = 0.547
		Gleason = 7: 10.4%	Gleason = 7: 9.0%	*p* = 1
		Gleason ≥ 8: 14.5%	Gleason ≥ 8: 10.8%	*p* = 0.564
		Very low-risk PCa: 3.5%	Very low-risk PCa: 3%	*p* = 1.000
Richard J. Bryant et al. [21]	Biopsy-naïve	Combined with targeted sampling TRUS Gleason Grade Group ≥ 2: 54%	Combined with targeted sampling LATP Gleason Grade Group ≥ 2: 60%	OR 1.32; 95% CI 1.03–1.70; *p* = 0.031
Maria Angela Cerruto et al. [19]	Biopsy-naïve	Detection rate (any Gleason grade): 46.29%	Detection rate (any Gleason grade): 44.44%	*p* = 0.846
Olivier Wegelin et al. [18]	Prior-negative biopsy	Cognitive-targeted: clinically significant PCa: 33%, any grade PCa: 43.6%	Fusion-targeted: clinically significant PCa: 34%, any grade PCa: 49.4%	For any Gleason score detection, differences are not statistically significant
		MRI-targeted: clinically significant PCa: 33%, any grade PCa: 54.5		

Abbreviations: TR, transrectal access; TP, transperineal access; PCa, prostate cancer; csPCa, clinically significant prostate cancer; SysPbx/Sys, systematic biopsy; FusPbx/Fus, fusion biopsy; FUS-TB, fusion-targeted biopsy uses combined US and MR images; comPbx, combined biopsy (combination of fusion and systematic biopsy); COG-TB, cognitive-targeted biopsy physician locates lesions based on memory/experience; MRI-TB, in-bore MRI-targeted biopsy; US, ultrasound; MR/MRI, magnetic resonance imaging; CDR, cancer detection rate; ISUP, International Society of Urological Pathology (grading scale); GGG, Gleason Grade. Note: The study by Borkowetz et al. [17] utilized a paired, within-patient design where patients underwent TP fusion-targeted biopsy combined with TR systematic biopsy. Readers should note that this protocol evaluates the access route (TP vs. TR) alongside differing targeting modalities (fusion vs. systematic) simultaneously. Wegelin et al. (FUTURE trial) [18] was included in this analysis to provide critical context regarding MRI-targeting technologies and procedural heterogeneity, despite the study’s primary objective not being a direct comparison of versus TR biopsy approaches.

**Table 3:** Comparison of prostate cancer detection efficacy for each access route, considering the malignant tumor’s localization within the prostate gland.

No.	Study Name	Lesion Location	TR Efficacy	TP Efficacy	Comment
[20]	Schieda et al.	Anterior zone (anterior lesions)	16% (95% CI 7–31%)	29% (95% CI 15–49%)	RR 1.81; 95% CI 1.05–3.12; *p* = 0.03–significant TP advantage
		Apex (apical lesions)	No data	No data	RR 0.91; 95% CI 0.60–1.37; *p* = 0.65–no significant difference between groups
		Base (basal lesions)	No data	No data	RR 0.72; 95% CI 0.45–1.14; *p* = 0.16–no significant difference between groups
[24]	Ploussard et al.	Posterior zone (posterior lesions)	59.0%	44.3%	*p* = 0.0443–significant TR advantage
		Anterior zone (anterior lesions)	26.5%	40.6%	*p* = 0.2228–no significance
		Peripheral zone (PZ)	Lower CCR	Higher CCR	No statistical significance
[19]	Cerruto et al.	Transitional zone (TZ)	shorter cores	longer cores	Better biopsy quality in TP; no statistical significance for CDR
		Apex	shorter cores	longer cores	More effective sampling in TP; no significance for CDR
		Mid prostate	Lower CCR	Higher CCR	Statistically insignificant differences
[18]	Wegelin et al.	Anterior zone	COG-TB, csPCa: 44% MRI-TB, csPCa: 35.7%	FUS-TB, csPCa: 48.6%	No statistical significance between methods; *p* = 0.6 for csPCa
		Posterior zone	COG-TB, csPCa: 26.1% MRI-TB, csPCa: 28.9%	FUS-TB, csPCa: 20%	No statistical significance between methods; *p* = 0.7 for csPCa
		Peripheral zone	COG-TB, csPCa: 27.3% MRI-TB, csPCa: 31.9%	FUS-TB, csPCa: 20.5%	No statistical significance between methods; *p* = 0.5 for csPCa
		Transition zone	COG-TB, csPCa: 50% MRI-TB, csPCa: 60%	FUS-TB, csPCa: 50%	No statistical significance between methods; *p* > 0.9

Abbreviations: TR, transrectal; TP, transperineal; PZ, peripheral zone; TZ, transitional zone; CCR, cancer core rate; CDR, cancer detection rate; csPCa, clinically significant prostate cancer; FUS-TB, fusion-targeted biopsy; COG-TB, cognitive-targeted biopsy; MRI-TB, magnetic resonance imaging-targeted biopsy. Note: Wegelin et al. (FUTURE trial) [18] was included in this analysis to provide critical context regarding MRI-targeting technologies and their diagnostic efficacy in specific prostate zones, despite the study’s primary objective not being a direct comparison of TP versus TR biopsy approaches.

**Table 4:** Prostate cancer detection efficacy depending on the biopsy type and access route.

Study (Ref. No.)	Biopsy Type	Detection Efficacy	Statistical Significance
Borkowetz et al. [17]	Combined (Fusion + Systematic)	Clinically significant PCa: 44%; Overall PCa: 52%	Clinically significant PCa: Combined vs. Fusion vs. Systematic: *p* < 0.005
	Transperineal Fusion	Clinically significant PCa: 38%; Overall PCa: 47%	Clinically significant PCa: Fusion vs. Systematic: *p* = 0.296; Overall PCa: Fusion vs. Systematic: *p* = 0.15
	Transrectal Systematic	Clinically significant PCa: 35%; Overall PCa: 43%	N/A
	Combined (Fusion + Systematic)	Clinically significant PCa: 61% (PI-RADS 4–5) vs. 14% (PI-RADS ≤ 3)	*p* < 0.005
Tricard et al. [23]	Combined Transperineal (Systematic + Targeted)	Clinically significant detection rate: 27.5%	Transperineal vs. Transrectal: *p* = 0.019
	Combined Transrectal (Systematic + Targeted)	Clinically significant detection rate: 7.7%	
Ploussard et al. [24]	Targeted Transperineal	Clinically significant PCa: 47.2%; Overall PCa: 71.3%	Clinically significant PCa: *p* = 0.6235; Overall PCa: *p* = 0.2209
	Targeted Transrectal	Clinically significant PCa: 54.2%; Overall PCa: 64.1%	
Ber et al. [27]	Transperineal Fusion	Clinically significant PCa: 41.6%; Overall PCa: 57.1%	Difference in clinically significant PCa: 15.6%; 90% CI: 3.2–27.9%; *p* = 0.029
	Transrectal Fusion	Clinically significant PCa: 26%; Overall PCa: 41.6%	
Guo et al. [28]	Transperineal vs. Transrectal	PCa (Gleason Grade Group 6, 7, 8): 35.3% vs. 31.9%	*p* = 0.566
Bryant et al. [21]	LATP with systematic and targeted biopsy	Gleason Grade Group ≥ 2: 60%	OR 1.32; 95% CI: 1.03–1.70; *p* = 0.031
	TRUS with systematic and targeted biopsy	Gleason Grade Group ≥ 2: 54%	N/A
Cerruto et al. [19]	Systematic Transrectal	Cancer Detection Rate: 46.29%	*p* = 0.846 (Not Significant)
	Systematic Transperineal	Cancer Detection Rate: 44.44%	
	Systematic Transperineal vs. Systematic Transrectal	21.43% positive cores vs. 16.79% positive cores	*p* = 0.022; however, no significant overall detection advantage for systematic Transperineal

Abbreviations: comPbx, combined prostate biopsy; FusPbx, fusion prostate biopsy; SysPbx, systematic prostate biopsy; csPCa, clinically significant prostate cancer; PCa, prostate cancer; PI-RADS, Prostate Imaging–Reporting and Data System; TP biopsy/TP, transperineal biopsy/transperineal; TR biopsy/TR, transrectal biopsy/transrectal; csCDR, clinically significant cancer detection rate; GGG, Gleason Grade Group; LATP, local anaesthetic transperineal; TRUS, transrectal ultrasound; CDR, cancer detection rate. Note: The study by Borkowetz et al. [17] utilized a paired, within-patient design where patients underwent TP fusion-targeted biopsy combined with TR systematic biopsy. Readers should note that this protocol evaluates the access route (TP vs. TR) alongside differing targeting modalities (fusion vs. systematic) simultaneously.

**Table 5:** Prostate cancer detection efficacy in various patient subgroups considering the applied access route.

Study (Ref. No.)	Patient Subgroup	Transrectal Efficacy	Transperineal Efficacy	Comment
Mian et al. [32]	Overall (all patients)	47% clinically significant PCa	43% clinically significant PCa	No information of statistical significance
	MRI-positive	56% clinically significant PCa	53% clinically significant PCa	N/A
	MRI-positive, biopsy-naïve	59% clinically significant PCa	62% clinically significant PCa	N/A
	Anterior tumors on MRI	44.5% clinically significant PCa	39% clinically significant PCa	N/A
	MRI-negative	16% clinically significant PCa	14% clinically significant PCa	N/A
Borkowetz et al. [17]	Biopsy-naïve patients	Systematic biopsy clinically significant PCa: 35%	Fusion biopsy clinically significant PCa: 38%	Combined biopsy (Systematic + Fusion) efficacy = 44%; Fusion vs. Systematic: *p* = 0.296 (Not Significant); Combined vs. single: *p* < 0.005 (significant)
Ploussard et al. [24]	Biopsy-naïve patients	Significant PCa: 54.2%, Overall detection rate: 64.1%	Significant PCa: 47.2%, Overall detection rate: 71.3%	*p* = 0.6235 (clinically significant cancer, ISUP ≥ 2 detection); *p* = 0.2209 (any grade detection)
Guo et al. [28]	Biopsy-naïve patients	Detection rate: 35.3%	Detection rate: 31.3%	No significant statistical difference between methods; *p* = 0.566
		Gleason ≤ 6: 10.4%	Gleason ≤ 6: 10.8%	*p* = 0.547
		Gleason = 7: 10.4%	Gleason = 7: 9.0%	*p* = 1
		Gleason ≥ 8: 14.5%	Gleason ≥ 8: 10.8%	*p* = 0.564
		Very low-risk PCa: 3.5%	Very low-risk PCa: 3%	*p* = 1.000
Bryant et al. [21]	Biopsy-naïve	Combined with targeted sampling TRUS Gleason Grade Group ≥ 2: 54%	Combined with targeted sampling LATP Gleason Grade Group ≥ 2: 60%	OR 1.32; 95% CI 1.03–1.70; *p* = 0.031
Cerruto et al. [19]	Biopsy-naïve	Detection rate (any Gleason): 46.29%	Detection rate: 44.44%	*p* = 0.846
Wegelin et al. [18]	Prior-negative biopsy	Cognitive-targeted: clinically significant PCa: 33%, any grade PCa: 43.6%	Fusion-targeted: clinically significant PCa: 34%, any grade PCa: 49.4%	For any Gleason score detection, differences are not statistically significant
		MRI-targeted: clinically significant PCa: 33%, any grade PCa: 54.5%		

Abbreviations: comPbx, combined prostate biopsy; FusPbx, fusion prostate biopsy; SysPbx, systematic prostate biopsy; csPCa, clinically significant prostate cancer; PCa, prostate cancer; PI-RADS, Prostate Imaging–Reporting and Data System; TP biopsy/TP, transperineal biopsy/transperineal; TR biopsy/TR, transrectal biopsy/transrectal; csCDR, clinically significant cancer detection rate; GGG, Gleason Grade Group; LATP, local anaesthetic transperineal; TRUS, transrectal ultrasound; CDR, cancer detection rate. Note: Studies by Borkowetz et al. [17] and Wegelin et al. (FUTURE trial) [18] were included to provide critical context regarding MRI-targeting technologies in specific patient subgroups. Readers should note that Borkowetz et al. [17] evaluated TP fusion versus TR systematic biopsy simultaneously, while Wegelin et al. [18] compared TP fusion against TR cognitive and in-bore MRI-targeted biopsies.

**Table 6:** Comparison of infectious complication rates.

Study Author	Sample Size (n)	TP Group (%)	TR Group (%)	*p*-Value
Schieda et al. [20]	233	0/119 (0%)	0/114 (0%)	1.00
Bryant et al. [21]	1126	Primary infections at 7 days: 1 (<1%) *	Primary infections at 7 days: 7 (1%) *	0.782
		Secondary infections at 7 days: 54 (10%) *	Secondary infections at 7 days: 72 (13%) *	0.094
		Primary infections at 4 months: 6 (1%) *	Primary infections at 4 months: 13 (2%) *	0.001
		Secondary infections at 4 months: 113 (20%) *	Secondary infections at 4 months: 120 (21%) *	0.601
Tricard et al. [23]	90	2 (4.4%) **	6 (13.3%) **	0.268 ***
Hu et al. [25]	567	0 (0%)	4 (1.4%)	0.124
Mian et al. [31]	718	2 (0.5%) ****	1 (0.3%) ****	1.00
Berquin et al. [29]	128	1 (1.5%)	2 (3.3%)	0.601

Abbreviations: TR, transrectal access; TP, transperineal access. * Percentage share in the structure of recorded complications in a given group. ** Total number of infections for two biopsy series (2 vs. 6 cases per group of 45). *** *p*-value for total infections in Tricard study calculated by Fisher’s test (2 vs. 6 cases per group of 45). **** Number of confirmed infectious events in Mian study.

**Table 7:** Comparison of characteristics and frequency of selected non-infectious complications.

Study Author (Sample Size)	Parameter	TP Group (n, %)	TR Group (n, %)	*p*-Value
Bryant et al. [21] (n = 1126)	Overall non-infectious complications	454 (81%)	436 (77%)	0.111
	Blood in stool (overall)	62 (11%)	174 (31%)	<0.001
	Blood in urine (visible)	23 (4.1%)	37 (6.6%)	0.064
	Blood in semen	42 (7%)	44 (8%)	0.757
	Acute urinary retention	35 (6%)	27 (5%)	0.358
Waisman et al. [30] (n = 782)	Blood in urine	291 (79%)	249 (69%)	0.002
	Blood in stool	34 (9%)	64 (18%)	0.001
	Blood in semen	193 (72%)	181 (70%)	0.564
	Voiding disorders	67 (23%)	43 (16%)	0.033
Mian et al. [31] (n = 718)	Overall non-infectious complications	8 (2.2%)	6 (1.7%)	0.589
	Acute urinary retention	1 (0.3%)	1 (0.3%)	1.00
Hu et al. [25] (n = 567/658 *)	Acute urinary retention	1 (0.3%)	3 (1.1%)	0.369
	Bleeding requiring intervention	0 (0.0%)	1 (0.4%)	1.00
Guo et al. [28] (n = 328)	Mild complications (overall)	75 (44.9%)	66 (41.0%)	0.473
	Major complications (overall)	1 (0.6%)	7 (4.3%)	0.037
	Mild rectal bleeding	0 (0.0%)	14 (8.7%)	<0.001
	Major rectal bleeding	0 (0.0%)	2 (1.2%)	0.241
	Mild hematuria	33 (19.8%)	37 (23.0%)	0.478
	Major hematuria	0 (0.0%)	0 (0.0%)	1.00
Ploussard et al. [24] (n = 250)	Complications ≥ Grade 2	46 (35.7%)	53 (40.5%)	0.4256
	Acute urinary retention	2 (1.5%)	4 (3.3%)	0.442
	Hematospermia median duration (days)	5.5 days	9.0 days	-
Schieda et al. [20] (n = 233)	Acute urinary retention	0 (0.0%)	1 (1.0%)	0.438
	Bleeding	0 (0.0%)	0 (0.0%)	1.00
Berquin et al. [29] (n = 128)	Hematuria after 12 h	49 (73.1%)	37 (60.0%)	0.146
	Hematuria after 24 h	41 (61.2%)	22 (36.1%)	0.005
	Hematuria after 48 h	31 (46.3%)	20 (32.8%)	0.126
	Rectal bleeding after 12 h	15 (22.4%)	23 (37.7%)	0.064
	Rectal bleeding after 24 h	9 (13.4%)	6 (9.8%)	0.591
	Rectal bleeding after 48 h	2 (3.0%)	5 (8.2%)	0.256
	Blood in semen after 12 h	5 (9.0%)	8 (13.1%)	0.564
	Blood in semen after 24 h	7 (10.4%)	6 (9.8%)	1.00
	Blood in semen after 48 h	15 (22.4%)	17 (27.9%)	0.480
Tricard et al. [23] (n = 90)	Acute urinary retention (after 2 series)	1 (2.2%)	2 (4.4%)	1.00
Cerruto et al. [19] (n = 108)	Overall non-infectious complications	7 (13%) **	7 (13%) **	1.00
	Urethral bleeding	5 (9.3%)	0 (0.0%)	0.056
	Rectal bleeding	0 (0.0%)	4 (7.4%)	0.118
	Acute urinary retention	0 (0.0%) **	1 (1.9%) **	1.00

Abbreviations: TR, transrectal access; TP, transperineal access. * Number of patients who completed the survey (approx. 80% of the original cohort). ** Recalculated for the entire group cohort (the source text gave overall % share of complications). *p*-values were calculated using Fisher’s or *χ*^2^ method.

**Table 8:** Base-case cost-effectiveness analysis results from the Huang et al. [26] PREVENT-based economic simulation.

Category	Parameter	TR	TP
Clinical parameters (7 days)	Probability of infection	1.6%	0%
	Probability of urinary retention	1.1%	0.3%
	Duration of post-procedural pain	—	1 h
Costs (USD/patient)	Fixed costs (MRI + biopsy)	2532.00	2532.00
	Disposable materials	57.03	218.89
	Prophylactic antibiotics	5.51	—
	Rectal swab	91.00	—
	Treatment of infection	134.67	0.00
	Treatment of urinary retention	19.67	5.33
	Total cost	2782.84	2699.22
Health effect (per patient)	Total utility (QALY)	0.0410033	0.0410738
Incremental analysis (TP vs. TR)	Δ Cost	—	−83.62 USD
	Δ QALY	—	+0.0000705
	ICER	—	Dominant strategy

Abbreviations: TR, transrectal biopsy; TP, transperineal biopsy; QALY, Quality-Adjusted Life Year; ICER, Incremental Cost-Effectiveness Ratio; MRI, magnetic resonance imaging; USD, United States dollar.

**Table 9:** Cost and health outcome analysis from the TRANSLATE within-trial economic analysis by Little et al. [22].

Category	Parameter	TR	TP	Difference (TP − TR)
Costs by observation period	0–7 days	506	681	+175 (149–201; <0.01)
	7–35 days	149	133	−14 (−48–20; 0.407)
	Day 35–4 months	294	260	−43 (−141–55; 0.388)
	Total cost (model-based)	917	1062	+149 (61–236; 0.001)
Costs by healthcare service category (0–4 months)	Hospital care	70	54	−16
	Outpatient visit	193	179	−14
	Primary care	23	23	0
	Antibiotics	0.31	0.21	−0.10
	Drugs and medical devices for urinary disorders	14.41	3.55	−10.86
	Analgesics	0.42	0.99	0.57
Health effect	QALY	0.284	0.282	−0.004 (−0.009 to 0.001; 0.098)
Incremental analysis (TP vs. TR)	Δ Cost	—	—	+£149 (95% CI: 61–236; *p* = 0.001)
	Δ QALY	—	—	−0.004 (95% CI: −0.009 to 0.001; *p* = 0.098)
	ICER	—	—	TP dominated by TR

Abbreviations: TR, transrectal biopsy; TP, transperineal biopsy; NHS, National Health Service; QALY, Quality-Adjusted Life Year; ICER, Incremental Cost-Effectiveness Ratio; CI, confidence interval; *p*, statistical significance level.

## Data Availability

No new data were created or analyzed in this study. Data sharing is not applicable to this article.

## Abbreviations

AUA, American Urological Association; AUR, Acute Urinary Retention; CCR, Cancer Core Rate; CDR, Cancer Detection Rate; CI, Confidence Interval; COG-TB, Cognitive-Targeted Biopsy; comPbx, Combined Prostate Biopsy; csCDR, Clinically Significant Cancer Detection Rate; csPCa, Clinically Significant Prostate Cancer; DRE, Digital Rectal Examination; EAU, European Association of Urology; FUS-TB, Fusion-Targeted Biopsy; FusPbx, Fusion Prostate Biopsy; GGG, Gleason Grade Group; HTA, Health Technology Assessment; ICER, Incremental Cost-Effectiveness Ratio; IPSS, International Prostate Symptom Score; ISUP, International Society of Urological Pathology; LATP, Local Anaesthetic Transperineal; MDR, Multi-Drug Resistant; mpMRI, Multiparametric Magnetic Resonance Imaging; MRI, Magnetic Resonance Imaging; MRI-TB, Magnetic Resonance Imaging-Targeted Biopsy; MUS, Micro-Ultrasound; NHS, National Health Service; NRS, Numerical Rating Scale; OR, Odds Ratio; PCa, Prostate Cancer; PI-RADS, Prostate Imaging–Reporting and Data System; PROMs, Patient-Reported Outcome Measures; PSA, Prostate-Specific Antigen; PV, Prostate Volume; PZ, Peripheral Zone; QALY, Quality-Adjusted Life Year; QoL, Quality of Life; RCT, Randomized Controlled Trial; RR, Relative Risk; SysPbx, Systematic Prostate Biopsy; TP, Transperineal; TR, Transrectal; TRUS, Transrectal Ultrasound; TZ, Transitional Zone; US, Ultrasound; USD, United States Dollar; VAS, Visual Analog Scale.
